# Alkaloid Profile, Anticholinesterase and Antioxidant Activities, and Sexual Propagation in *Hieronymiella peruviana* (Amaryllidaceae) †

**DOI:** 10.3390/plants14020281

**Published:** 2025-01-19

**Authors:** Olimpia Llalla-Cordova, Javier E. Ortiz, Luciana R. Tallini, Laura Torras-Claveria, Jaume Bastida, Lorena Celina Luna, Gabriela E. Feresin

**Affiliations:** 1 Instituto de Biotecnología, Facultad de Ingeniería, Universidad Nacional de San Juan, Av. Libertador General San Martín 1109 (O), San Juan CP5400, Argentina; ollallac@unam.edu.pe (O.L.-C.); jortiz@unsj.edu.ar (J.E.O.); lorenaluna@unsj-cuim.edu.ar (L.C.L.); 2 Consejo Nacional de Investigaciones Científicas y Técnicas (CONICET), CCT CONICET San Juan, Av. Libertador General San Martín 1109 (O), San Juan CP5400, Argentina; 3 Faculdade de Farmácia, Universidade Federal do Rio Grande do Sul, Av. Ipiranga 2752, Porto Alegre 90610-000, RS, Brazil; ruscheltallini@ub.edu; 4 Departament de Biologia, Sanitat i Medi Ambient, Facultat de Farmàcia i Ciències de l’Alimentació, Universitat de Barcelona, Av. Joan XXIII 27-31, 08028 Barcelona, Spain; lauratorrascl@ub.edu (L.T.-C.); jaumebastida@gmail.com (J.B.)

**Keywords:** Amaryllidaceae, Hieronymiella, anti-oxidant, germination, cholinesterases, Peru

## Abstract

*Hieronymiella peruviana*, a recently described endemic species from southern Peru, belongs to the Amaryllidaceae family and is known for its diversity of alkaloids. Amaryllidoideae have been studied for their diverse biological activities, particularly for their properties in treating neurodegenerative diseases. This work examines the alkaloidal profile using GC-MS and UPLC-MS/MS of alkaloid-enriched extracts obtained from the leaves and bulbs of *H. peruviana* and their inhibitory activity against acetylcholinesterase (AChE) and butyrylcholinesterase (BuChE) enzymes. In addition, the phenolic and flavonoid content in the methanolic extract from bulbs was quantified and the antioxidant capacity was evaluated. Moreover, the seed germination was investigated under four temperature conditions (15, 20, 25, and 30 °C). Twenty-two alkaloids, most of them of the homolycorine- and galanthamine-type, including galanthamine, were identified in the alkaloid extracts by means of GC-MS and UPLC-MS/MS analysis. Lycorine and 8-*O*-Demethylhomolycorine were the most abundant alkaloids in the bulbs and leaves, respectively. The leaves and bulbs alkaloid extracts demonstrated strong AChE inhibition (IC_50_ = 5.20–8.60 µg/mL) and moderate BuChE inhibition (IC_50_ = 90.20–122.76 µg/mL). The bulbs’ methanolic extract exhibited mild antioxidant activity, showing 2,2-diphenyl-1-picrylhydrazyl (DPPH) and ferric reducing antioxidant power (FRAP) values of 16.36% at 500 μg/mL and 58.31 mg Trolox equivalents (TE)/g, respectively. Seed germination was most effective at 20 °C and 25 °C. Finally, 60 days after germination, the survival rate of *H. peruviana* seedlings was 48.33%. These findings establish *H. peruviana* as a promising source of bioactive alkaloids with potential pharmacological and therapeutic applications, as well as providing critical insights for its propagation and conservation.

## 1. Introduction

The Amaryllidoideae subfamily (from Amaryllidaceae family) produces alkaloids that exhibit significant biological activities, including significant anti-inflammatory, anticancer, and antiviral effects [1]. They are mostly notable for the inhibition of acetylcholinesterase (AChE) and butyrylcholinesterase (BuChE) enzymes, crucial for acetylcholine hydrolysis in the synaptic cleft [2]. Inhibiting these enzymes is a key strategy in palliative neurodegenerative diseases treatment, like Alzheimer’s disease (AD), by increasing acetylcholine levels in the brain and enhancing cholinergic neurotransmission [3].

Among the most studied alkaloids, galantamine (Gal), approved since 2001 by the FDA for the treatment of early-stage of AD, is a long-acting, selective, reversible, and competitive AChE inhibitor [4]. However, the increasing demand for Gal exceeds the production capacity from natural sources like *Narcissus* spp., *Galanthus* spp., and *Leucojum aestivum* [5]. Therefore, it is essential to search for alternative sustainable sources for the production of cholinesterase inhibitors.

**Figure 1:**
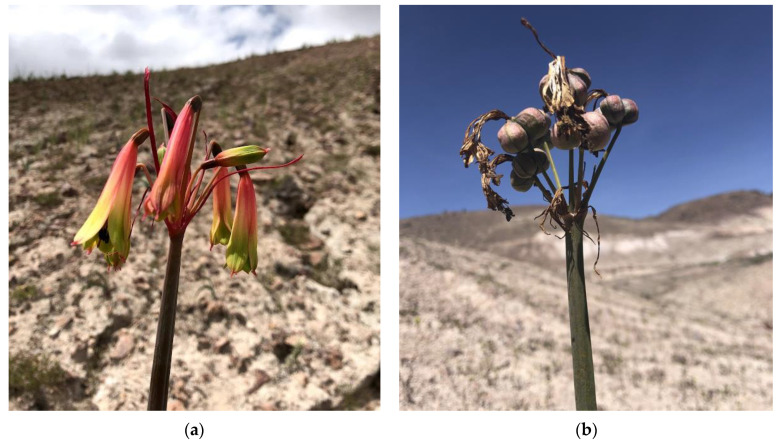
*Hieronymiella peruviana* analyzed in this study. (**a**) Inflorescence; (**b**) Plants in fruiting stage in their natural habitat.

The genus *Hieronymiella* (Amaryllidaceae, tribe Eustephieae) comprises approximately nine species, distributed mainly in the Andes, from south-central Bolivia to northwestern and central Argentina. *H. peruviana* was recently recorded in southern Peru [6]. This species, endemic to the Department of Moquegua, is characterized by winged staminal filaments with crisscrossed sickle-shaped appendages and its adaptation to semi-arid pumice soils (Figure 1). Although no traditional uses have been documented for *H. peruviana*, the genus *Hieronymiella* has been recognized as a potential source of alkaloids due to its chemical diversity. Gal has been identified in *H. aurea*, *H. caletensis*, *H. clidanthoides*, *H. marginata*, and *H. speciosa*. Additionally, sanguinine and clidanthine have been isolated from this genus, both demonstrating strong AChE and BuChE inhibition [7].

Alkaloid profiles of Amaryllidaceae are typically analyzed using gas chromatography-mass spectrometry (GC-MS), detecting volatile compounds; however, UPLC-ESI-MS technique identifies polar and heat-sensitive compounds that may be degraded during GC-MS analysis, providing complementary and unique information about the alkaloids in the extract [8]. A recent extensive review has revealed that the Amaryllidoideae subfamily possesses non-alkaloid metabolites with promising bioactive properties. These include phenolic compounds and flavonoids, recognized as natural antioxidants essential for reducing oxidative stress [9].

Developing propagation methods is crucial for species within the Amaryllidoideae subfamily, such as *Hieronymiella*, for conservation and sustainable utilization as well as to ensure sufficient biomass for extracting bioactive compounds. In this regard, seed propagation is more efficient and cost-effective than bulb division or micropropagation [10]. However, knowledge about the seed germination requirements for many Amaryllidaceae species is still limited, especially regarding the effect of the temperature over germination [11]. *Hieronymiella peruviana* was recently described as a new endemic species from Peru, and no chemical profile nor biological activities have been reported until now. The aim of this work was to study the chemical profile and evaluate the anticholinesterases and antioxidant activities, as well as the germination conditions, for *H. peruviana* propagation.

## 2. Results and Discussion

### 2.1. Alkaloid Profile

After extraction was complete, the AEE yield was 0.20% for bulbs and 0.21% for leaves. The alkaloids content of these extracts was analyzed by GC-MS. A total of twenty-two alkaloids were detected in *H. peruviana* (bulbs and leaves), including seven not identified compounds. Some of these molecules were tentatively classified as homolycorine-type structures according to their fragmentation pattern (Table 1).

**Figure 2:**
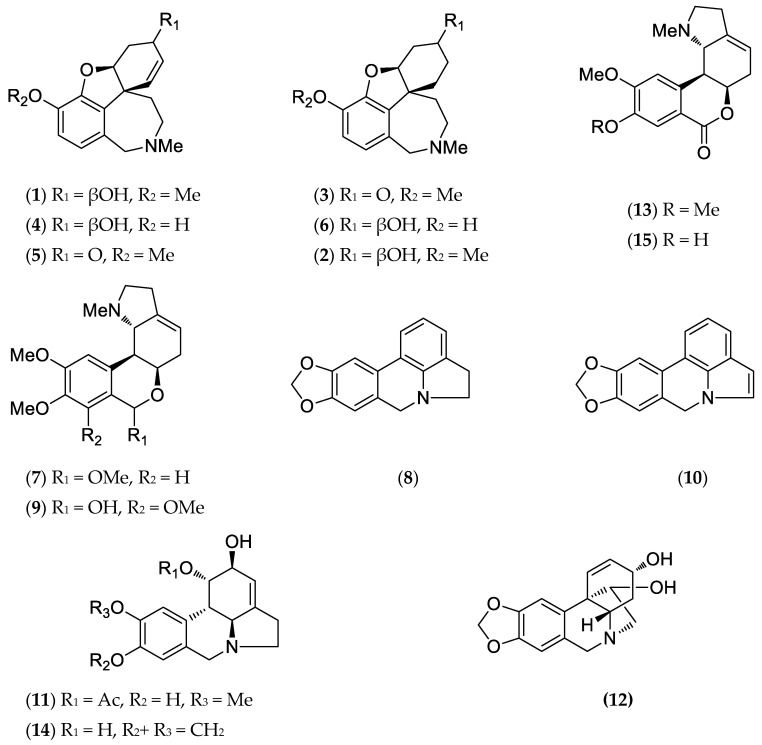
Alkaloids structures from *H. peruviana.*

The identified alkaloids (Figure 2) were classified according to Berkov et al. [1].

The proportion of each compound in the alkaloid extract was expressed as a percentage of the total ion current (TIC), and their relative percentages, based on Total Ion Current (TIC), are presented in Table 1. These data do not express quantification, although they can be used to compare the relative abundances of each component. The results obtained were analyzed using AMDIS 2.64 software and the NIST database. Compounds were identified by comparing their mass spectral patterns and retention indices with the data recorded in the literature [1].

**Figure 3:**
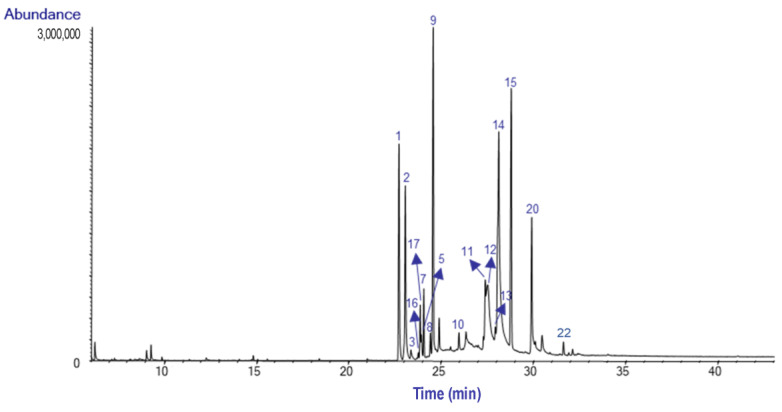
GC MS chromatogram of the alkaloid extract from the bulbs of *Hieronymiella peruviana.*

**Figure 4:**
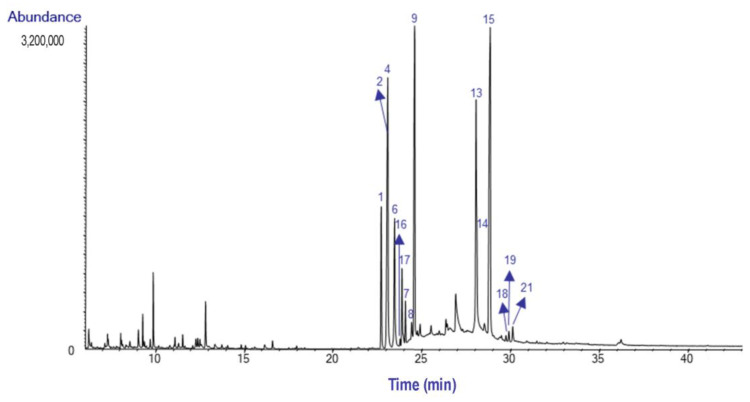
GC-MS chromatogram of the alkaloid extract from the leaves of *H. peruviana.*

The GC-MS chromatograms of the AEE from the bulbs and leaves of *H. peruviana* are presented in Figure 3 and Figure 4, respectively.

This is the first alkaloid profile reported for the species *Hieronymiella peruviana*. The AEE of bulbs and leaves showed a similar alkaloid composition, characterized by a prevalence of homolycorine- and galanthamine-type alkaloids. These findings suggest that *H. peruviana* is an important source of alkaloids with this structural type. The homolycorine -type skeleton comprises 80 known structures, underscoring its chemodiversity and its potential role in bioactive compound discovery [1]. The diagnostic ion m/z 109, characteristic of homolycorine-type alkaloids with a Δ^3,4^ double bond and no substitution at C2, was observed in the spectra [12].

The most abundant alkaloid was lycorine (**14**), with a relative abundance of 21.1% TIC in the bulb, while 8-*O*-Demethylhomolycorine (**15**) was the most abundant in the leaves, with 24.4% TIC. Lycorine has been reported in *Hieronymiella aurea*, *H. caletensis*, *H. clidanthoides*, *H. marginata*, and *H. speciosa*, with relative abundances ranging from 3.83% to 92.98% TIC, as documented by Ortiz et al. [7]. However, Ortiz et al. did not report the presence of 8-*O*-Demethylhomolycorine in the bulbs of these species [7]. This alkaloid has also been identified in *Narcissus tazetta*, where it was primarily concentrated in flowers and leaves [13].

Nerinine (**9**) was identified in both bulbs and leaves of *H. peruviana*. This finding is particularly noteworthy, as Nerinine has not been previously reported in *Hieronymiella* species from Argentina, and its occurrence in other Amaryllidoideae species is limited.

Sanguinine (**4**) was identified in *H. peruviana* leaves with a relative abundance of 14.9% TIC. This finding contrasts with previous studies on *H. marginata*, where sanguinine was less abundant.

Gal (**1**) stands out, accounting for 10% TIC in the bulbs and 5.6% TIC in the leaves. Similar findings have been reported in the Argentina species, with TIC values ranging from 0.1% to 9.79%, although its abundance varies depending on the species and geographic location. In this regard, Gal has been identified in *Hieronymiella aurea*, *H. caletensis*, *H. clidanthoides*, *H. marginata*, and *H. speciosa*, as reported by Ortiz et al. (2018) [7].

Lycoramine (**2**) was identified in the bulbs and leaves of *H. peruviana* with a relative abundance of 9.8 and 12.3% TIC, respectively. In comparison, *H. speciosa* exhibited the highest relative abundance (29.80% TIC), while *H. marginata* showed significantly lower levels, with only 0.85% TIC [7].

To better understand the chemical composition of the AEE of *H. peruviana*, an UPLC-MS/MS analysis was performed. The results showed the presence of the [M+H^+^]^+^ for all the alkaloids identified in the GC-MS analysis. Coincidentally, most of the identified alkaloids in the GC-MS analysis were also identified in the UPLC-MS/MS chromatogram, however the relative abundance of each alkaloid showed differences between both methods. Additionally, the UPLC-MS/MS analysis displayed mass values [M+H^+^]: (334, 368, 336, 362) that were not found in GC-MS. These results are in agreement with previously reported data indicating that EI and ESI ionization/fragmentation differed considerably in the analysis of all the Amaryllidaceae alkaloid-types, especially for the detection of homolycorine-type or dinitrogenous-type alkaloids [14]. Thus, the UPLC-MS/MS data are complementary to those of GC-MS, providing additional information regarding the chemical composition of the AEE of *H. peruviana*.

The alkaloids identified in *H. peruviana* possess significant bioactive properties. Gal, known for its role as an AChE and BuChE inhibitor, also exhibits antimicrobial, antioxidant, and anticancer activities [15]. Lycorine is a weak cholinesterase inhibitor, and its structure, with specific modifications, could serve as a scaffold for developing more potent AChE inhibitors [16]. Additionally known for its broad pharmacological profile, lycorine exhibits antiviral, anti-inflammatory, antifibrotic, antibacterial, hepatoprotective, antioxidant, anticancer, and selective cytotoxic activities, as well as the ability to inhibit ascorbic acid synthesis [17]. Lycoramine and other alkaloids have recently attracted attention for their therapeutic potential in skin cancer by selectively reducing melanoma cell viability and UVB-induced ROS and IL-6 production in keratinocytes [18].

According to ProTox 3.0 predictions, 8-*O*-demethylhomolycorine exhibits moderate toxicity with an LD50 of 1190 mg/kg (Class 4), making it less toxic at higher doses compared to Gal (LD50 = 85 mg/kg, Class 3). However, its low AChE inhibition activity (0.60 vs. 0.93 for Gal) limits its potential for direct neurodegenerative applications. The absence of carcinogenic and mutagenic activities suggests favorable genetic safety, providing a foundation for future research into derivatives with enhanced therapeutic profiles. For nerinine, ProTox 3.0 predicts limited potential in neurodegenerative applications. Nerinine demonstrates moderate toxicity (LD50 = 765 mg/kg, Class 4), low hepatotoxicity (0.90), and low neurotoxicity (0.67). However, its elevated immunotoxicity (0.98) indicates the need for caution in prolonged applications.

### 2.2. Cholinesterase Inhibitory Activity

The AEE from *H. peruviana* bulbs exhibited strong AChE inhibitory activity (IC_50_: 8.60 ± 0.37 µg/mL) and moderate BuChE inhibition (IC_50_: 122.76 ± 0.37 µg/mL). The leaves’ AEE showed similar AChE and BuChE IC_50_ values (5.20 ± 0.53 and 90.20 ± 0.87 µg/mL, respectively), indicating selective inhibition towards AChE. This selectivity aligns with findings in Argentinean *Hieronymiella* species, which demonstrated strong AChE inhibition (IC_50_: 1.84–15.40 µg/mL) and weaker BuChE inhibition (IC_50_: 23.74 to >200 µg/mL) [7]. The cholinesterase inhibitory activity observed in *H. peruviana* can be attributed primarily to galantamine-type alkaloids, which include Gal (**1**), Lycoramine (**2**), Lycoraminone (**3**), Sanguinine (**4**), Narwedine (**5**), and *O*-Demethyllycoramine (**6**), as identified in the alkaloid profile. The higher inhibition observed in the leaves could be mainly due to Sanguinine (**4**), which has been identified as a potent AChE inhibitor [7].

Despite their high relative abundance, Nerinine, Lycorine, and 8-*O*-Demethylhomolycorine show limited contributions to the observed cholinesterases inhibition. Lycorine’s low activity is likely due to the absence of free hydroxyl groups at positions C1 and C2, which are associated with improved enzyme binding in lycorine-type alkaloids [19]. In addition, 8-*O*-Demethylhomolycorine and Nerinine are predicted to have minimal effects, with ProTox 3.0 scores of 0.60 for both compounds.

However, the interaction between different alkaloids can enhance or reduce their inhibitory effects on these enzymes. A similar study on Amaryllidaceae extracts suggests that combining various alkaloids, even at low concentrations, enhances inhibition [20]. These findings highlight the selective AChE inhibitory potential of *H. peruviana*.

### 2.3. Total Phenolic, Flavonoid Contents and Antioxidant Activity

The concentration of TPC (Table 2) in *H. peruviana* is higher compared to *Galanthus transcaucasicus* (3.41 to 4.45 mg GAE/g) in bulb, flower, and root reported by Karimi et al. [21]. Additionally, Boshra et al. [22] reported, in *Narcissus pseudonarcissus*, a low TPC (1.29 mg GAE/g) and higher TFC (1.19 mg QE/g) compared to those found in *H. peruviana*.

The TPC in *H. peruviana* significantly contributes to its antioxidant activity, considering the low TFC. The FRAP result for *H. peruviana* indicates higher antioxidant activity compared to *Allium lycaonicum* (18.34 mg TE/g) [23]. In contrast, the EtOH extract of *Pancratium maritimum* bulbs showed higher TPC (60.9 mg CAE/g) and TFC (24.6 mg QE/g) and greater antioxidant activity than *H. peruviana* [24].

For the evaluated concentrations in DPPH (1 to 500 μg/mL), no increasing trend in antioxidant capacity was observed, with values ranging from 13.19% to 16.36%. Consequently, the IC_50_ could not be determined. However, the activity (17.50% DPPH) was comparable to the EtOH extract of *N. pseudonarcissus*, even at a lower concentration (0.5 vs. 1 mg/mL) [22]. Khalifa [25] reported superior antioxidant activity in the EtOH extract of *Hippeastrum vittatum* (IC_50_: 285.20 μg/mL) in the DPPH assay, despite its relatively lower TPC (10.48 mg GAE/g). Higher antioxidant activity was also observed in *G. transcaucasicus*.

Antioxidant activity can also possibly be influenced by alkaloids such as Gal, lycorine, and nerinine. These alkaloids have shown antioxidant properties. Nevertheless, under certain conditions or at higher concentrations, they may act as prooxidants, generating reactive oxygen species (ROS) and causing oxidative stress [26]. This dual behavior may influence the observed antioxidant activity in the ME of *H. peruviana.*

### 2.4. Seed Propagation

The effect of four temperatures (15, 20, 25, and 30 °C) on the germination of *H. peruviana* seeds stored for three months at −5 °C was evaluated. The germination variables of *H. peruviana* varied in response to different temperatures, particularly germination energy (GE), germination speed index (GSI), mean germination time (MGT), and median germination time (T_50_), as shown in Table 3.

The GP (%) was high, reaching 94% at 20 °C and decreasing to 82% at 30 °C. No statistically significant differences were observed. There is no information in the literature for *Hieronymiella* seeds germination, and limited information for others of the Amaryllidoideae genus, such as *Zephyranthes*, *Rhodophiala*, *Habranthus*, and *Narcissus*. Santa Cruz et al. [27] observed the same in *Zephyranthes mesochloa*, where 30 °C resulted in a decrease in GP (%), with values between 94% and 75%.

Similarly, in *Rhodophiala bifida*, it was reported that temperatures up to 28 °C favored germination above 90%; however, at 33 °C, there was a marked decrease in germination, accompanied by an increase in seed mortality [28].

**Figure 5:**
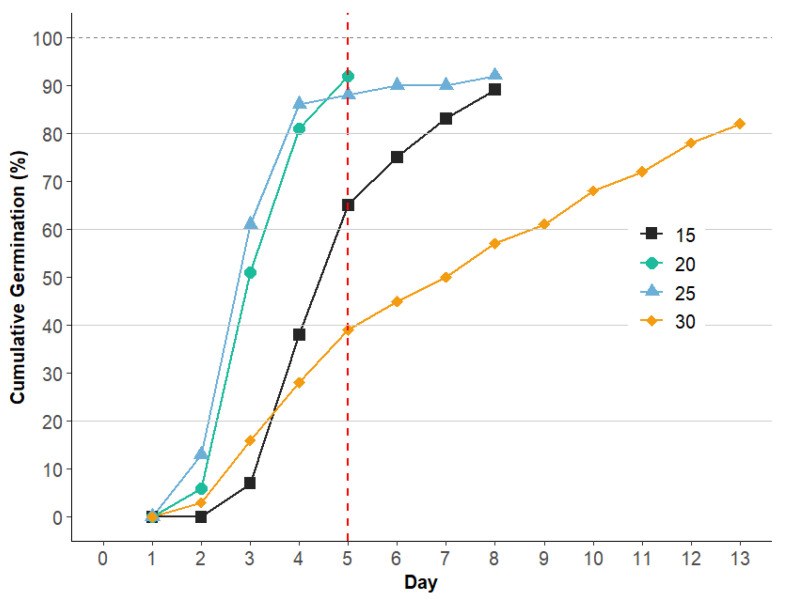
Cumulative germination percentage of *H. peruviana* seeds over a 13-day period at 4 temperatures (15 °C, 20 °C, 25 °C, and 30 °C). The dotted red line at day 5 indicates the day used for calculating germination energy (GE) based on early germination accumulation. Different symbols indicate temperatures, showing rapid germination and stabilization at 20 °C and 25 °C, while 15 °C results in a more moderate rate and 30 °C shows a slower progression of germination.

The progression of germination varied across temperatures (Figure 5). At 20 °C and 25 °C, germination accumulated rapidly in the early days, while at 15 °C, the trend was moderate in comparison. Conversely, at 30 °C, the accumulation rate was more gradual, indicating reduced germination efficiency at this temperature.

The analysis of GE, calculated up to day 5 (Figure 5) to allow for uniform comparison across temperatures, revealed high GE at 20 °C and 25 °C, reaching 92% and 88%, respectively, indicating strong initial vigor under these conditions. At 15 °C, the GE of *H. peruviana* was 65%, whereas at 30 °C, it decreased to 39%. A similar trend was observed in *Narcissus radicandorum*, where germination was negatively affected by elevated temperatures, with a very low GE (1%) at 28/14 °C [29]. These findings suggest that high temperatures may adversely affect the germination energy in these species.

The Germination Speed Index (GSI) was significantly higher at 25 °C (5.97) and 20 °C (5.61) compared to the value observed in *Habranthus cardenasianus* (5.20) [10]. Oliveira et al. [30] indicated that higher GSI values reflect stronger seed vigor. These results indicate that intermediate temperatures enhance germination speed.

Lower T_50_ values at 20 °C and 25 °C indicate faster and more efficient germination compared to extreme temperature such as 15 °C and 30 °C, where delays occurred. These findings align with GSI values, indicating that intermediate temperatures promote rapid germination in *H. peruviana*, while higher and lower temperatures reduce germination speed.

**Figure 6:**
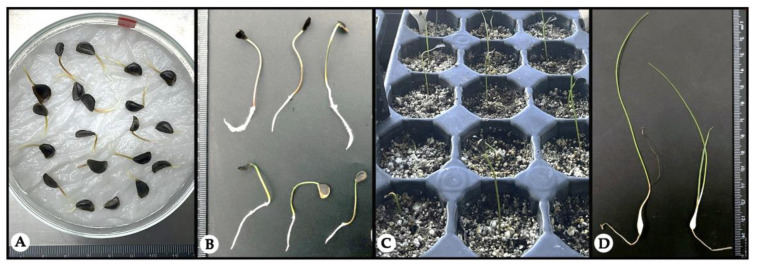
(**A**) Germination of seeds; (**B**) Germinated seeds with radicles and hypocotyls; (**C**) Seedlings transplanted into substrate trays; (**D**) Seedlings with developed bulbs after 60 days**.**

Seedlings germinated at 20 °C and 25 °C (a total of 120) were selected for the evaluation of survival, as shown in Figure 6. After germination, the seedlings developed a primary (true) leaf measuring between 10 and 14 cm, and the diameter of the formed bulbils ranged from 0.20 to 0.35 cm. The survival percentage in *H. peruviana* was lower (48.33%) compared to the 63% survival rate reported by Salazar et al. [31] for *Rhodophiala pratensis* over the same period. Post-germination survival is a critical challenge for many species [32], highlighting the importance of evaluating factors such as light and substrate management to optimize establishment and growth.

## 3. Materials and Methods

### 3.1. Chemicals

Sulfuric acid (H_2_SO_4_) was purchased from Merck Química Argentina (Buenos Aires, Argentina). Commercial Folin-Ciocalteu (FC) reagent, 2,2-Diphenyl-1-picrylhydrazyl (DPPH), ferric chloride hexahydrate, 2,4,6-tris(2-pyridyl)-s-triazine, trolox, quercetin, and gallic acid (GA) were purchased from Sigma-Aldrich.

AChE from *Electrophorus electricus* (electric eel), BuChE from equine serum, potassium phosphate (K_2_HPO_4_), sodium dihydrogen phosphate (NaH_2_PO_4_), sodium chloride (NaCl), 5,5′-dithio-bis-(2-nitrobenzoic acid) (DTNB), acetylthiocholine iodide (ATC), butyrylthiocholine iodide (BTC), and Gal were obtained from Sigma-Aldrich.

### 3.2. Plant Material and Procedure Extraction

Leaves and bulbs of *Hieronymiella peruviana* Huaylla, Slanis & Llalla [6] were collected in March 2021, during the flowering period at Jaguay Chico (16°56′02′′ S, 70°53′25′′ W), General Sánchez Cerro Province, Moquegua Department, Peru.

Capsules of *H. peruviana* were collected in April 2024 and the seeds were obtained after full capsules opened and natural seed were released. The seeds were manually sorted based on size, uniform shape, and color. The selected seeds were stored at −5 °C until the start of germination trials. The collected material was identified and deposited in the Herbarium Moqueguensis of the Universidad Nacional de Moquegua, Peru (voucher specimen: MOQ- 1168).

Fresh bulbs (183 g) and leaves (397 g) were cut into small pieces and then dried under an air current at 40 °C until a constant weight was reached, which required approximately 5 days. The dried material was then coarsely ground to a granular form, resulting in 38.52 g of dried bulbs and 35.77 g of dried leaves. Each sample was separately subjected to maceration with MeOH, and was kept in darkness at room temperature for 72 h. The maceration process was performed twice for both bulbs and leaves. The extracts were then filtered, and the solvent was removed using a rotary evaporator at 38 °C, yielding the methanolic extracts (ME). Subsequently, a portion of the ME was used to obtain an alkaloid-enriched extract following the method described by Ortiz et al. (2023) [8], with some modifications.

The ME was dissolved in a 2% (*v*/*v*) H_2_SO_4_ and the pH was adjusted between 3.5 and 4. Three extractions with ethyl ether (Et_2_O) (3 × 100 mL) were performed to remove fatty matter. The remaining aqueous phase was alkalinized to pH 9–10 with 20% (*w*/*v*) NaOH and subjected to extractions with ethyl acetate (AcOEt) (3 × 100 mL) to recover the alkaloids. The basic AcOEt solution was separated, and anhydrous sodium sulphate was added to remove any remaining water. Finally, the solvent was evaporated to dryness using a rotary evaporator in order to obtain the alkaloid-enriched extract (AEE). The percentage yield was calculated, and the sample was stored for subsequent analyses.

### 3.3. Gas Chromatography Coupled to Mass Spectrometry (GC-MS) and Ultra Performance Liquid Chromatography (UPLC) Analysis

The AEE of *H. peruviana* were analyzed by GC-MS, using an equipment model 6890/MSD 5975 (Hewlett Packard, Palo Alto, CA, USA) operating in electron impact ionization mode (70 eV). Compound separation was carried out with a DB-5 MS column (30 m × 0.25 mm × 0.25 µm). The temperature program was as follows: from 100 °C to 180 °C at a rate of 15 °C/min, with a 1-min hold at 180 °C; followed by an increase from 180 °C to 300 °C at 5 °C/min, maintaining this temperature for 1 min. The injector temperature was set at 280 °C, with helium flow as carrier gas at 0.8 mL/min and a split ratio of 1:20.

The data were processed with AMDIS 2.64 software. Compounds were identified by comparing mass spectra and retention index (RI) values compared with a library of previously characterized Amaryllidaceae alkaloids held in the Natural Products Laboratory University of Barcelona [33] and with the NIST 20 database (NIST Mass Spectral Database, PC-Version 2020, National Institute of Standardization and Technology, Gaithersburg, MD, USA). Relative abundance of each compound in the alkaloid-enriched extract (AEE) was expressed as a percentage of the total ionic current (TIC). These data do not express quantification, although they can be used to compare the relative abundances of each component.

The LC-MS analysis was performed in an ACQUITY H–Class UPLC instrument equipped with a XEVO TQ-S micro triple quadrupole mass spectrometer (Waters Corp, Milford, MA, USA) with electrospray ionization (ESI). An UPLC ACQUITY BEH C18 (1.7 μm, 2.1 mm × 100 mm) column was used for separation at 35 °C. The mobile phase con-sisted of A (0.1% formic acid), B (acetonitrile, 0.1% formic acid), and C (methanol) with a flow rate of 0.2 mL/min. The gradient conditions were as follows: initially, 95%A–5%B and hold for 2 min; 5 min, 85%A–15%B; 10 min, 80%A–10%B–10%C and hold for 7 min; 18 min, 95%A–5%B and hold for 2 min; completing 20 min. The AAE *H. peruviana* were prepared at 100 ppm. The samples were dissolved in a mixture of methanol:water (50:50) and filtered through a membrane filter (0.22 µm). The injection volume was 10 µL. The capillary, cone, and collision energies were 2 kV, 43 V, and 30 eV, respectively. The data were acquired in ESI positive mode, MS2 scan function (50–1000 Da), and processed using MassLynx Software V4.2 (Waters, Milford, MA, USA). This combination of techniques enabled a comprehensive qualitative and semi-quantitative profiling of the metabolites in the samples, facilitating the precise identification of the alkaloids of interest.

### 3.4. Cholinesterase Inhibitory Activities

The inhibitory activity of AChE and BuChE enzymes was evaluated following the method of Ellman et al. (1961) [34] with modifications. In each well of a 96-well microplate, 50 µL of AChE or BuChE enzyme solution (0.25 U/mL, in phosphate buffer saline (PBS): 8 mM K_2_HPO_4_, 2.3 mM NaH_2_PO_4_, 0.15 M NaCl, pH 7.5) and 50 µL of the AAE *H. peruviana* dissolved in the same buffer were added. The plates were incubated at room temperature for 30 min. Subsequently, 100 µL of the substrate solution, containing DTNB and ATC or BTC (0.6 mM), prepared in a saline solution with Na_2_HPO_4_ (pH 7.5), was added. The absorbance at 405 nm was recorded using a Thermo Scientific Multiskan FC spectrophotometer, 5 min after the reaction began. To calculate the IC_50_ values, the concentrations of the AAE *H. peruviana* assayed ranged from 1 to 200 µM. Gal was used as a positive control and PBS was used as a negative control. The enzyme-inhibitory activity was calculated as a percentage compared to an assay using a PBS without inhibitor. IC_50_ values were expressed as the mean ± standard deviation (SD) of three individual determinations, each performed in triplicate. The enzyme-inhibitory data were analyzed with the software package Prism 10.4.1 (Graph Pad Inc., San Diego, CA, USA).

### 3.5. Preliminary Toxicological Assessment

The ProTox 3.0 web tool was employed to predict the toxicological profiles of the compounds, including hepatotoxicity, carcinogenicity, immunotoxicity, mutagenicity, cytotoxicity, LD50, and acetylcholinesterase (AChE) inhibition. The web server (https://comptox.charite.de/protox3/ accessed on 3 November 2024) [35] functions as a virtual laboratory for predicting the toxicological properties of small molecules. This platform was utilized to evaluate the potential toxicological characteristics of alkaloids with limited prior studies.

### 3.6. Assessment of Total Phenolic Content (TPC) and Total Flavonoid Content (TFC)

TPC was determined using the method described by Helrich et al. [36] with modifications. In a 96-well microplate, 10 µL of ME, 12.5 µL of diluted Folin–Ciocalteu reagent, and 37.5 µL of 20% (*w*/*v*) Na_2_CO_3_ were added. Reaction mixture was incubated for 30 min at room temperature in dark followed by taking absorbance at 750 nm using a Multiskan FC microplate reader (Thermo Scientific, Waltham, MA, USA). The calibration curve was constructed using GA at concentrations of 0, 0.15, 0.3, 0.6, 1.2, and 2.35 mM. Results were expressed as milligrams of GA equivalents per gram of methanolic extract (mg GAE/g ME).

The trichloride aluminum (AlCl_3_) colorimetric method was employed to assess the TFC, based on the protocol by Ismail et al. [37] with modifications. In each well, 125 µL of ME and 125 µL of 2% (*w*/*v*) AlCl_3_ were added. The solution was left to stand for 10 min at room temperature. Absorbance was measured at 450 nm using a microplate reader (Thermo Scientific, USA). The calibration curve was constructed using quercetin standard solutions at concentrations of 0, 0.03, 0.07, 0.15, 0.22, and 0.30 mM. Results were expressed as milligrams of quercetin equivalents per gram of methanolic extract (mg QE/g ME). Values obtained in triplicate for TPC and TFC are reported as mean ± standard deviation (SD).

### 3.7. Antioxidant Assays

#### 3.7.1. DPPH Radical Scavenging Activity

Total antioxidant content was determined using a method based on the protocol by Luna et al. [38] with modifications. A solution of DPPH (1,1-diphenyl-2-picrylhydrazyl) radical in MeOH was used for the evaluation. Five different concentrations of ME (1, 10, 50, 100, and 500 μg/mL) were prepared. Then, 150 μL of DPPH was added to 75 μL of each sample in a 96-well plate. The solution was left to stand in the dark for 5 min at room temperature, after which the absorbance was measured at 517 nm using a Thermo Scientific Multiskan FC microplate reader (Waltham, MA, USA). Different concentrations of Quercetin (20–120 mg/mL) were used as standard to obtain the calibration curve. The percentage of discoloration (free radical scavenging capacity) was calculated using the following formula:

DPPH scavenging capacity (%) = [1 − ((A_s_ − A_c_)/A_DPPH_)] × 100 (1)

where A_c_ represents the control absorbance, As represents the absorbance of the tested extract, and A_DPPH_ the absorbance of DPPH radical.

#### 3.7.2. Ferric Reducing Antioxidant Property Assay (FRAP)

The ferric reducing antioxidant power (FRAP) assay was conducted following the protocol described by Benzie and Strain [39] with modifications [40]. Five concentrations of the ME (1, 10, 50, 100, and 500 μg/mL) were prepared. For the assay, 10 µL of each extract or Trolox standard (1 mM) was added to 190 µL of the FRAP solution, consisting of 300 mM acetate buffer (pH 3.6), 10 mM TPTZ (2,4,6-tripyridyl-s-triazine) dissolved in 40 mM HCl, and 20 mM FeCl_3_·6H_2_O in a 10:1:1 ratio. After 30 min of incubation, the absorbance was measured at 595 nm using a Multiskan FC microplate reader (Thermo Scientific, Waltham, MA, USA). Several concentrations of Trolox at concentrations of 0 to 1 mM were used as standard to plot calibration curve. Results were expressed as milligrams of quercetin equivalents per gram of methanolic extract (mg QE/g ME).

### 3.8. Germination and Seedling Survival

Seeds stored for three months were disinfected with 1% sodium hypochlorite for 5 min and rinsed 3 times with distilled water. Germination was evaluated under four temperature treatments (15 °C, 20 °C, 25 °C, and 30 °C), with five replicates per treatment. Each replicate consisted of 20 seeds, resulting in a total of 100 seeds per treatment. The seeds were placed in Petri dishes lined with filter paper moistened with distilled water and incubated in germination chambers (Ingelab I-291D model) under a 12 h light/12 h dark photoperiod. The moisture of the filter paper was maintained by periodically adding distilled water. Germination was monitored daily over a 13-day period, and seeds were considered germinated when a radicle of ≥3 mm was observed.

The germination percentage (GP) was calculated as the proportion of germinated seeds relative to the total seeds sown, expressed as a percentage.

GP = (Number of seeds germinated/Total seeds sowed) × 100%(2)

Germination energy (GE) was measured as the cumulative percentage of germinated seeds up to the day of maximum germination speed, which in this case was day 5, and was calculated as:

GE% = (Cumulative number of germinated seeds up to day 5/Total seeds sowed) × 100%(3)

The Germination Speed Index (GSI) or germination velocity was calculated using the following formula [41]:

GSI = G_1_/T_1_ + G_2_/T_2_ + … + G_k_/T_k_ (4)

G_1_, G_2_, …, G_k_ represent the number of seeds germinated at time T_1_,T_2_,…,T_k_, respectively.

Mean times germination (MTG) was calculated using the following equation [42]:

MTG = ∑(n × d)/∑n(5)

where, n is the number of seeds germinated in each day, d is the number of days since the beginning of germination, ∑n is the total number of germinated seeds at the end of the experiment. The median germination time (T_50_), representing the time required for 50% of the seeds to germinate, was determined using a formula adapted from Farooq et al. [43]:

T_50_ = *ti* + ((*N*/2 − *ni*)(*tj − ti*))/(*nj* − *ni*) (6)

where *N* is the final number of germinated seeds. *ti* and *tj* are the days immediately before and after reaching 50% cumulative germination (*N*/2). *ni* and *nj* are the cumulative numbers of seeds germinated on days *ti* and *tj*, respectively, with *ni* < *N*/2 < *nj*.

The radicles of germinated seedlings were transplanted into plastic trays with dimensions of 4.9 cm in diameter and 9 cm in height. The trays were filled with a substrate composed of perlite, coarse sand, and peat in a proportion of 5:3:2. The seedlings were maintained under controlled conditions at 18–22 °C with a 10/14-h light/dark photoperiod. Survival was monitored over a 60-day period and calculated as the percentage of living seedlings relative to the total transplanted seedlings.

### 3.9. Statistical Data Analysis

The dataset for AChE, BuChE, TPC, TFC, DPPH, and FRAP assays consisted of triplicate measurements, with results expressed as mean ± standard deviation (SD). For germination experiments, one-way ANOVA was performed, and Tukey’s post-hoc test was applied to identify significant differences (*p* < 0.05). Data were analyzed using RStudio (version 2024.04.2, Build 764). Calibration curves for TPC, TFC, DPPH, and FRAP assays were plotted in Excel.

## 4. Conclusions

The profile of the alkaloid-enriched extract of *H. peruviana* exhibited general consistency between bulbs and leaves, with differences regarding the relative abundances of several of their alkaloids. Lycorine is the most abundant compound in bulbs, while 8-*O*-demethylhomolycorine predominates in leaves. The alkaloid extracts demonstrate strong and selective inhibitory activity against acetylcholinesterase (AChE), attributed to the presence of galanthamine-type alkaloids. The methanolic extract exhibits mild antioxidant activity, driven by its phenolic compound content. The optimal temperatures for germination indicate favorable conditions for sexual propagation, supporting future conservation and sustainable use strategies for research focused on this and other *Hieronymiella* species. The presence of unidentified alkaloids in *H. peruviana* highlights its potential as a source of bioactive compounds, emphasizing the need for further phytochemical and pharmacological studies.

## Figures and Tables

**Table 1:** Alkaloids profile of *Hieronymiella peruviana* by GC-MS. Values are expressed as a relative percentage of TIC.

Alkaloids (Alkaloid-Type)		M^+^	RI	Bulbs	Leaves
(**1**)	Galanthamine (Gal-T)	287	2397.6	10.0	5.6
(**2**)	Lycoramine (Gal-T)	289	2419.6	9.8	12.3
(**3**)	Lycoraminone (Gal-T)	287	2439.2	0.3	-
(**4**)	Sanguinine (Gal-T)	273	2420.9	-	14.9
(**5**)	Narwedine (Gal-T)	285	2475.7	0.8	-
(**6**)	O-Demethyllycoramine (Gal-T)	275	2477.4	-	6.4
(**7**)	O-Methyllycorenine (Hom-T)	331	2484.1	2.4	1.3
(**8**)	Anhydrolycorine (Lyc-T)	251	2507.2	1.1	0.7
(**9**)	Nerinine (Hom-T)	347	2517.6	19.9	17.6
(**10**)	11,12-dehydroanhydrolycorine (Lyc-T)	249	2610.4	0.9	-
(**11**)	Sternbergine (Lyc-T)	331	2707.7	3.2	-
(**12**)	Hamayne (Hae-T)	331	2715.1	1.1	-
(**13**)	Homolycorine (Hom-T)	315	2755.1	2.0	8.3
(**14**)	Lycorine (Lyc-T)	287	2759.1	21.1	4.6
(1**5**)	8-O-Demethylhomolycorine (Hom-T)	301	2838.2	16.9	24.4
(**16**)	Not identified (Hom-T*a) ‘	301*b	2464.8	0.2	0.2
(1**7**)	Not identified (Hom-T*a) ‘	329*b	2471.6	2.0	2.8
(**18**)	Not identified (Hom-T*a) ‘	345*b	2850.4	-	0.2
(**19**)	Not identified	281*b	2865.3	-	0.4
(**20**)	Not identified	279*b	2875.4	7.7	-
(**21**)	Not identified	343*b	2876.2	-	0.4
(**22**)	Not identified	293*b	3017.8	0.6	-

M^+^: molecular ion; RI: retention index; *a possible alkaloid type skeleton; *b possible molecular weight. Gal-T: galanthamine-type; Hom-T: homolycorine-type; Lyc-T: lycorine-type; Hae-T: haemanthamine-type.

**Table 2:** Evaluation of the total phenolic content (TPC), total flavonoid content (TFC), and antioxidant activity (DPPH scavenging capacity and FRAP) in extracts from bulb of *H. peruviana*.

TPC ^a^ (mg GAE/g)	TFC ^b^ (mg QE/g)	DPPH ^c^ (%)	FRAP ^d^ (mg TE/g)
12.89 ± 0.27	0.28 ± 0.03	16.36 ± 0.20	58.31 ± 13.70

^a^ Total phenolic content (TPC) expressed as mg gallic acid/g of methanolic extract (ME). ^b^ Total flavonoid content (TFC) expressed as mg quercetin/g of methanolic extract (ME). ^c^ Antiradical DPPH activities are expressed % at 500 μg/mL concentration. ^d^ Expressed as gr trolox equivalents/g of methanolic extract (ME).

**Table 3:** Effect of temperature on germination percentage, germination energy, speed index, and mean germination time of *H. peruviana.*

Temperature	GP (%)	GE (%)	GSI	MGT (days)	T_50_ (days)
15 °C	92 ± 10.4 ^a^	65 ± 12.7 ^b^	3.88 ± 0.55 ^b^	5.13 ± 0.28 ^b^	4.31 ± 0.32 ^b^
20 °C	94 ± 5.48 ^a^	92 ± 5.70 ^a^	5.61 ± 0.29 ^a^	3.55 ± 0.21 ^c^	2.95 ± 0.22 ^bc^
25 °C	93 ± 5.70 ^a^	88 ± 5.70 ^a^	5.97 ± 0.42 ^a^	3.41 ± 0.18 ^c^	2.71 ± 0.13 ^c^
30 °C	82 ± 4.47 ^a^	39 ± 8.94 ^c^	3.19 ± 0.22 ^b^	6.67 ± 1.03 ^a^	5.92 ± 1.63 ^a^

Values are mean ± standard deviation. The different superscripts (a, b, c) indicate significant differences between temperatures (*p* < 0.05) according to Tukey’s test.

## Data Availability

The data presented in this study are openly available in at DOI reference number.
